# The Anti-inflammatory Protein TSG-6 Regulates Chemokine Function by Inhibiting Chemokine/Glycosaminoglycan Interactions*

**DOI:** 10.1074/jbc.M116.720953

**Published:** 2016-04-04

**Authors:** Douglas P. Dyer, Catherina L. Salanga, Scott C. Johns, Elena Valdambrini, Mark M. Fuster, Caroline M. Milner, Anthony J. Day, Tracy M. Handel

**Affiliations:** From the ‡ Skaggs School of Pharmacy and Pharmaceutical Sciences, University of California, San Diego, La Jolla, California 92093-0684,; the § Institute of Infection, Immunity and Inflammation, College of Medical, Veterinary and Life Sciences, University of Glasgow, Glasgow G12 8TA, Scotland, United Kingdom,; the ¶ Medical and Research Sections, Veterans Affairs San Diego Healthcare System, La Jolla, California 92093,; the ‖ Department of Medicine, Division of Pulmonary and Critical Care, University of California, San Diego, La Jolla, California 92093, and; the ** Wellcome Trust Centre for Cell-Matrix Research,; ‡‡ Faculty of Life Sciences, University of Manchester, Manchester M13 9PT, United Kingdom

**Keywords:** cell migration, chemokine, glycosaminoglycan, heparan sulfate, heparin-binding protein, surface plasmon resonance (SPR), chemokine-binding protein, TNF-stimulated gene 6 (TSG-6)

## Abstract

TNF-stimulated gene-6 (TSG-6) is a multifunctional protein secreted in response to pro-inflammatory stimuli by a wide range of cells, including neutrophils, monocytes, and endothelial cells. It has been shown to mediate anti-inflammatory and protective effects when administered in disease models, in part, by reducing neutrophil infiltration. Human TSG-6 inhibits neutrophil migration by binding CXCL8 through its Link module (Link_TSG6) and interfering with the presentation of CXCL8 on cell-surface glycosaminoglycans (GAGs), an interaction that is vital for the function of many chemokines. TSG-6 was also found to interact with chemokines CXCL11 and CCL5, suggesting the possibility that it may function as a broad specificity chemokine-binding protein, functionally similar to those encoded by viruses. This study was therefore undertaken to explore the ability of TSG-6 to regulate the function of other chemokines. Herein, we demonstrate that Link_TSG6 binds chemokines from both the CXC and CC families, including CXCL4, CXCL12, CCL2, CCL5, CCL7, CCL19, CCL21, and CCL27. We also show that the Link_TSG6-binding sites on chemokines overlap with chemokine GAG-binding sites, and that the affinities of Link_TSG6 for these chemokines (*K_D_* values 1–85 nm) broadly correlate with chemokine-GAG affinities. Link_TSG6 also inhibits chemokine presentation on endothelial cells not only through a direct interaction with chemokines but also by binding and therefore masking the availability of GAGs. Along with previous work, these findings suggest that TSG-6 functions as a pluripotent regulator of chemokines by modulating chemokine/GAG interactions, which may be a major mechanism by which TSG-6 produces its anti-inflammatory effects *in vivo*.

## Introduction

TSG-6 (TNF-stimulated gene/protein 6) is an inflammation-associated protein that has been shown to be up-regulated by pro-inflammatory mediators such as IL-1, TNF, and LPS in a broad range of cells and in the context of inflammatory diseases (1–6). It is an ∼35-kDa secreted protein composed of Link and CUB_C domains with an additional short N-terminal sequence (5, 7–10). Although initially found at high levels in the joints of patients with rheumatoid and osteoarthritis, suggesting a pro-inflammatory role (1), administration of TSG-6 was found to inhibit damage in inflammatory models, including arthritis (11–14) and transplant rejection (15), suggesting it possesses anti-inflammatory properties. TSG-6 has also been identified as a key mediator of anti-inflammatory effects of human mesenchymal stem cells in models of myocardial infarction (4), corneal damage (16), peritonitis (17), traumatic brain injury (18), acute lung injury (19), wound healing (20), and type 1 diabetes (21). One mechanism underlying its protective effects is thought to be its ability to inhibit the influx of neutrophils to inflammatory sites and the concomitant neutrophil-induced damage (4, 16, 18, 22, 23). To understand the basis for the protective effect of TSG-6, the Link module (Link_TSG6) was expressed in isolation (24, 25) and shown to reproduce the effects of the full-length protein in inhibiting neutrophil migration (26) and in binding to a number of ligands, including the glycosaminoglycans (GAGs)^4^ heparin and heparan sulfate (HS) (27, 28); moreover, Link_TSG6 inhibited rolling and transendothelial migration of neutrophils as determined by intravital microscopy (29). Link_TSG6 was also shown to interact with CXCL8 and to inhibit its presentation on and transport across endothelial cells, as well as its ability to recruit neutrophils (30), providing at least a partial explanation for the anti-inflammatory effects of TSG-6.

CXCL8 is a member of the chemotactic cytokine (chemokine) family of proteins, which are best known for their roles in regulating cell migration. They mediate cell recruitment by signaling through chemokine receptors on leukocyte cell surfaces (31–34). However, in addition to activating these G protein-coupled signaling receptors on migrating cells, chemokines interact with cell-surface GAGs (35–40). GAGs/proteoglycans are found in the extracellular matrix (ECM) (41) and ubiquitously on essentially all cell surfaces, including endothelial cells where they comprise a key component of the glycocalyx (41–44). The interaction of chemokines with GAGs enables their cell surface localization and facilitates formation of chemotactic gradients to guide leukocytes to sites of infection and inflammation (40). Moreover, GAG binding has been shown to be integral to the function of a number of chemokines, including CXCL8 (38, 39), CXCL12 (37), CCL2 (35), CCL5 (35), CCL7 (36), and CCL21 (40).

Given the prior observation that TSG-6 inhibits binding of CXCL8 to heparin and endothelial cell surfaces resulting in down-regulation of CXCL8-mediated neutrophil migration (30), we hypothesized that TSG-6 might inhibit GAG-mediated cell surface presentation of other chemokines that recruit different cell types (45). This hypothesis was motivated by the fact that in addition to neutrophils, TSG-6 administration results in reduced infiltration of other cell types during inflammation, including monocytes (4), T cells, and dendritic cells (47).

In this study, we demonstrate the ability of Link_TSG6 to interact with a wide range of chemokines from the CC and CXC subfamilies. Furthermore, we show that Link_TSG6 interacts with the GAG-binding region of these chemokines and inhibits their presentation on endothelial surfaces. These TSG-6/chemokine interactions are of particular interest given the lack of soluble chemokine-binding proteins identified in humans and other vertebrates, despite many having been identified in ticks, parasites, and viruses (48–50). Moreover, although chemokines play an integral role in the regulation of inflammation, their pharmaceutical targeting has proved largely unsuccessful, and such binding proteins could have therapeutic potential (51, 52).

## Experimental Procedures

### 

#### 

##### Protein Production and Purification

WT Link_TSG6 and the mutant Link_TSG6_T (K55A/K69A/K76A) (numbered as in the pre-protein throughout (7)) were expressed in *Escherichia coli* and refolded/purified as described previously (24, 25, 27). Biotinylated and WT chemokines CXCL4, CXCL12, CCL2, CCL5, CCL7, CCL19, CCL21, and CCL27 and associated mutants, where CCL21 Δ*CT* relates to residues 1–79 as described previously (53), were expressed and purified from *E. coli* as described previously (54–56).

##### Surface Plasmon Resonance (SPR)

In all instances, a BIAcore 3000 instrument (GE Healthcare) was used to generate binding curves. Analyte was flowed over the chip surface in running buffer (10 mm HEPES, 150 mm NaCl, 0.05% Tween 20 (v/v), pH 7.4) at varying concentrations for 5 min at 40 μl/min; subsequently, running buffer alone was flowed over the bound ligand and a nonspecific control surface for 5 min at 40 μl/min to monitor the dissociation phase of the interaction. Curves were then corrected with subtraction of nonspecific and buffer alone signals and analyzed with the BIAevaluation software (GE Healthcare) using the 1:1 Langmuir interaction model. The degree of fit to this model was assessed by using the χ^2^ values, where χ^2^ <10 was accepted as a good fit. In the instances where the χ^2^ value was significantly higher than 10 and visual inspection of the data suggested poor fitting, alternative models were used to fit the data (bivalent analyte or two-state reaction models); however, in no instances did these models improve the fit to the raw data. Given the less than ideal fitting for some datasets involving chemokines with Link_TSG6, the calculated affinities are considered “apparent affinities,” but they still allow for relative ranking of the interactions. These difficulties arise from the propensity of certain chemokines to oligomerize, as described previously (57).

##### SPR Analysis of Chemokine Binding to Immobilized Link_TSG6

The Link_TSG6 surface was generated on a C1 chip (GE Healthcare) as described previously (30). Briefly, the surface was activated with 100 μl of a 1:1 mix of NHS (0.1 m) and 1-ethyl-3-(3-dimethylaminopropyl)carbodiimide (0.2 m) before flowing over Link_TSG6 (20 μg/ml) in immobilization buffer (10 mm HEPES, pH 7.4) at 20 μl/min until the desired immobilization level was reached (800–1000 response units). Remaining active sites on the chip surface were blocked with 1 m ethanolamine (120 μl). The surface was then washed with 1 m NaCl followed by regeneration buffer (50 mm NaOH). Results from replicate chemokine injections before and after surface regeneration and at various times throughout the use of a given chip were used to monitor surface integrity; the data were highly reproducible indicating that the Link_TSG6 surface was unaffected by the regeneration treatment and remained stable throughout the experiments. Interaction analysis was undertaken as described above with a number of different chemokines and associated mutants; any ligand remaining bound to the Link_TSG6 surface was fully removed with regeneration buffer (160 μl) prior to the analysis of a different ligand.

##### SPR Analysis of Link_TSG6 Binding to Immobilized Heparin

A heparin surface was generated on a C1 chip as described previously (56). First, neutravidin was covalently immobilized to the surface until saturation using the 1-ethyl-3-(3-dimethylaminopropyl)carbodiimide/NHS chemistry described above. The surface was then washed extensively to remove non-covalently bound neutravidin before biotinylated unfractionated porcine intestinal heparin (Calbiochem) (0.2 mg/ml in 100 mm sodium acetate, pH 5.5) was flowed over the surface at 10 μl/min, until saturation was reached. SPR analysis was carried out as described above to determine the affinity of Link_TSG6 for immobilized heparin, using the same approach described previously to analyze chemokine/heparin interactions (56). Regeneration buffer was used following each cycle of chemokine injection and interaction analysis to clean the chip surface.

##### Chemokine/Heparin Interactions in Solid Phase Binding Assays

Solid phase binding assays were undertaken as described previously (30). Briefly, CCL2, CCL7, CCL19, or CXCL11 (250 nm) was immobilized onto Nunc MaxiSorp plates (ThermoScientific) in coating buffer (20 mm Na_2_CO_3_, pH 9.6) for 16 h at room temperature. Wells were then rinsed using assay buffer (10 mm NaOAc, 150 mm NaCl, 2% (v/v) Tween 20, pH 6.0) and blocked with assay buffer containing 5% (w/v) BSA at 37 °C for 90 min. Biotinylated heparin (made from 4th International Standard (58)) was then added at increasing concentrations (0–100 ng/well), and for competition binding assays, biotinylated heparin (25 ng/well) was added in combination with a range of Link_TSG6_T concentrations (0–1000 nm) in assay buffer at room temperature for 4 h. Plates were washed with assay buffer, and the level of bound heparin was then assessed by addition of ExtrAvidin-alkaline phosphatase (1:10,000) (Sigma) and subsequent incubation with detection reagent (Sigma*FAST p-*nitrophenyl phosphate solution (Sigma)).After 5 min of development, absorbance levels (405 nm) were taken and signal corrected against blank wells (no coated chemokine); in cases where differential background of coated/uncoated wells led to negative corrected values (*i.e.* at high Link_TSG6_T concentrations), these were assigned a value of 0. Data were analyzed and fit to the non-linear regression one-site binding model (GraphPad Prism Version 5.0) to provide an estimate of the IC_50_ values.

##### Chemotaxis and Transendothelial Migration

Chemotaxis experiments were undertaken using a 5-μm pore Transwell system (Corning Inc.) as described previously (56). Here, CCL19 or CCL21 (50 nm) was pre-incubated alone or in combination with different molar ratios of Link_TSG6 (1:2 or 1:1, Link_TSG6:chemokine) for 30 min at 37 °C, 5% CO_2_ in 600 μl of Dulbecco's modified Eagle's medium (DMEM) containing 10% fetal bovine serum (FBS) in the bottom chamber of the Transwell. L1.2 cells induced to express CCR7 or CCR5 (by incubation with 5 mm sodium butyrate for 18 h), or Jurkat cells expressing CXCR4, were resuspended in DMEM containing 10% FBS before addition to the top chamber of the Transwell apparatus (100 μl of 2 × 10^6^ cells/ml). Wells were incubated for 2 h at 37 °C, 5% CO_2_ before the suspended membranes were removed, and the cells in the bottom chamber (migrated cells) were counted using a Guava EasyCyte 8HT flow cytometer (EMD Millipore). For transendothelial migration experiments, EaHY926 human umbilical vein endothelial cells (100 μl of 1 × 10^6^ cells/ml) were coated onto a suspended Transwell membrane overnight in DMEM containing 10% FBS. The surface of the endothelial cells was washed with 100 μl of DMEM and aspirated before the experiment was undertaken as described above for chemotaxis experiments.

##### Endothelial/Collagen Chemokine Presentation Assay

These assays were undertaken using a similar method to that described previously (56). Specifically, a clear-bottomed black-walled 96-well polystyrene plate (Corning Inc.) was coated with 200 μl of 100 μg/ml type I collagen (Purecol, Advanced BioMatrix) for 1 h at 37 °C. EaHY926 human umbilical vein endothelial cells were added to each well (200 μl of 0.1 × 10^6^ cells/ml (20,000 cells/well) in DMEM containing 10% FBS) and incubated for 18 h (until confluent); this and all subsequent cellular incubations were carried out in 5% CO_2_ at 37 °C. Non-adherent cells were removed by washing with DMEM followed by two washes with PBS supplemented with 1 mm CaCl_2_ and 0.5 mm MgCl_2_ (cPBS). Alternatively, assays were undertaken on a collagen-coated surface alone. Biotinylated chemokine (CXCL4, CXCL12, and CCL21) or unlabeled chemokine (CCL2, CCL5, and CCL7) was incubated alone or in combination with different molar ratios of Link_TSG6 or heparin octasaccharide (dp8 (Neoparin); dp = degree of polymerization) in cPBS for 30 min at 37 °C before being added on top of the washed endothelial monolayer and incubated for 1 h. Alternatively, Link_TSG6 (500 nm) was pre-incubated on top of the washed endothelial cells for 30 min followed by three washes with cPBS and subsequent incubation of chemokine with endothelial cells for 1 h. Chemokine/Link_TSG6 solutions were then aspirated and the monolayers washed three times with cPBS for 2 min, before fixation of cells with 150 μl of ice-cold 4% (w/v) paraformaldehyde in PBS for 20 min at room temperature. These and subsequent washes/incubations were carried out with gentle rocking. The fixative agent was removed, and the endothelial cells were washed (four incubations of 4 min) using PBS + 0.05% Tween 20 before addition of 150 μl of blocking solution (LI-COR Biosciences) to each well and incubation for 90 min at room temperature. The solution was aspirated, and in the case of biotinylated chemokine, detection was undertaken with 100 μl of blocking solution containing streptavidin conjugated to IRDye 800CW biotin detection reagent (LI-COR Biosciences; 1:1000), which was added to each well and incubated for 90 min at room temperature. For non-labeled chemokine detection, 100 μl of blocking solution containing antibodies against CCL2, CCL5, or CCL7 (R&D Systems, 1 μg/ml) was added to each well and incubated for 1 h at room temperature. Cells were then washed four times (4 min) with 100 μl of PBS + 0.05% Tween 20, before addition of anti-goat IgG 800CW conjugate (1:5000) (LI-COR Biosciences) secondary antibody in blocking solution for 1 h at room temperature. In all cases, the solution was aspirated from the endothelial cells, which were then finally washed with 100 μl of PBS + 0.05% Tween 20 (four incubations of 4 min), followed by bound chemokine detection using an Odyssey imaging system (LI-COR Biosciences).

##### Cell Adhesion Assay

Murine bone marrow-derived dendritic cells (BMDCs) were cultured in DC media (RPMI 1640 medium, 10% FBS, 1% penicillin/streptomycin, 2 mm l-glutamine, 10 mm HEPES, 1 mm non-essential amino acids, and 55 μm β-mercaptoethanol) in the presence of 20 ng/ml recombinant murine GM-CSF (PeproTech) for 10 days. On day 9, BMDCs were treated with 200 ng/ml lipopolysaccharide (LPS 055:B5 (Sigma)) for 24 h. One day prior to the assay, murine lymphatic endothelial cells (SV-LECs, Alexander Laboratory, LSU Cell Culture Repository, Louisiana State University) were seeded onto a clear-bottomed, black-walled 96-well plate (Falcon) at 6.75 × 10^3^ cells/well. Prior to use, BMDCs were stained with calcein AM (eBioscience) for 1 h. Murine CCL21 (Biolegend) alone or in the presence of a 1:1 molar eq of Link_TSG6 was diluted in serum-free DMEM, incubated at room temperature for 30 min, then added to the SV-LECs, and incubated for an additional 30 min at 37 °C, 5% CO_2_. Following chemokine incubation on the SV-LECs, medium was removed, and 1 × 10^4^ of calcein-labeled BMDCs were added to each well. After 5 min, cell fluorescence was measured on a plate reader (DTX880, Beckman Coulter) to determine the maximum fluorescence for each well. Wells were then promptly washed two times with PBS to remove unbound BMDCs and read again to determine the extent of BMDC adhesion for each condition.

## Results

### 

#### 

##### TSG-6 Link Module Binds to Multiple Chemokines

**FIGURE 1.**
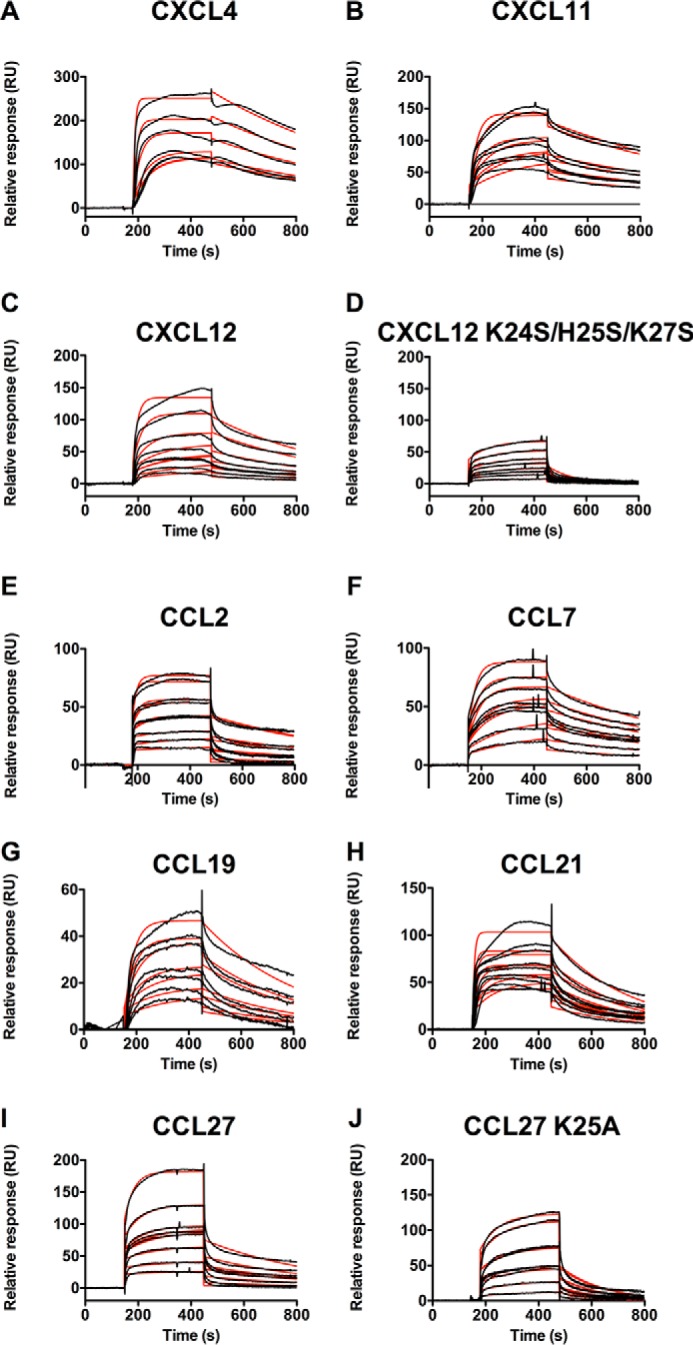
**Multiple chemokines bind to immobilized Link_TSG6.** Link_TSG6 was immobilized onto a BIAcore C1 chip, and different chemokines were passed over in running buffer at various concentrations to generate affinity estimates. Experimental curves are plotted (*black lines*) with fits (*red lines*) generated from a 1:1 Langmuir interaction model using analytes at a range of concentrations to generate “on” (*k_a_*) and “off” (*k_d_*) rates for the interaction and overall affinity (*K_D_* = *k_d_*/*k_a_*). *A,* CXCL4 (400, 200, 100, 50, 40, and 40 nm). *B,* CXCL11 (200, 150, 100, 75, and 50). *C,* CXCL12 (400, 200, 100, 50, 40, 40, 25, 12.5, and 6.25 nm). *D,* CXCL12 K24S/H25S/K27S (200, 150, 100, 75, 50, 37.5, 37.5, 25, and 12.5 nm). *E,* CCL2 (1000, 750, 500, 400, 250, 250, 200, 100, and 50 nm). *F,* CCL7 (1000, 750, 500, 400, 250, 250, 200, 100, 50, and 25 nm). *G,* CCL19 (200,150, 100, 75, 50, 37.5, and 25 nm). *H,* CCL21 (200, 150, 100, 75, 50, 37.5, 37.5, 25, and 12.5 nm). *I,* CCL27 (1000, 500, 400, 250, 250, 200, 100, and 50 nm). *J,* CCL27 K25A (1000, 750, 500, 400, 250, 250, 200, 100, and 50 nm). *RU,* response units.

Having previously established that Link_TSG6 inhibits CXCL8-mediated transendothelial migration of neutrophils via interactions with CXCL8, we set out to investigate whether it also interacts with other chemokines, explaining its ability to inhibit the migration of diverse cell types (4, 47) and to produce anti-inflammatory effects (5, 8). For these experiments, we used SPR with Link_TSG6 immobilized on an SPR chip and passed chemokines over the surface at varying concentrations. The rates of association (*k_a_*) and dissociation (*k_d_*) were determined from the sensorgrams and used to calculate dissociation constants (*K_D_* = *k_d_*/*k_a_*) with a 1:1 Langmuir interaction model. Interestingly, the SPR data revealed that Link_TSG6 binds to 10 different chemokines (Table 1 and Fig. 1), including CXCL4, CXCL11, CXCL12, CCL2, CCL7, CCL19, CCL21, and CCL27 (in addition to the known interactions with CXCL8 and CCL5 (30)) with *K_D_* values ranging from 1 to 85 nm. The chemokines fall into three major groups where CXCL4 (3.9 nm), CXCL11 (5.2 nm), CCL5 (1.9 nm), and CCL21 (4.8 nm) comprise a high affinity group (1–5 nm); CXCL8 (21 nm) (30), CXCL12 (15 nm), CCL2 (29.4 nm), CCL7 (18.4 nm), and CCL19 (17.5 nm) have slightly lower affinities (15–30 nm); and CCL27 showed the weakest interaction (85.2 nm). A fourth category is also apparent as Link_TSG6 has previously been shown to have little, if any, affinity for CXCL1 or CCL3 (30).

**TABLE 1:** **Surface plasmon resonance analysis of chemokine and mutant affinities for a Link_TSG6-coated surface** Rates of association (*k_a_*) and rates of dissociation (*k_d_*) were used to calculate overall affinity (*K_D_* = *k_d_*/*k_a_*) values for chemokine binding to immobilized Link_TSG6. The quality of the fit to a 1:1 Langmuir model is given by χ^2^, and the *R*_max_ (response units (RU)) values were calculated by application of this model, where χ^2^ <10 or χ^2^ <10% of *R*_max_ is indicative of a good fit. Data are representative of two independent experiments.

Chemokine	*k_a_*	*k_d_*	*K_D_*	χ^2^	*R*_max_ (RU)
	*m*^−*1*^ s^−*1*^	*s*^−*1*^	*nm*		
CXCL4	3.6 × 10^5^	1.4 × 10^−3^	3.9	52.9	270
CXCL8*^a^*	1.9 × 10^4^	3.9 × 10^−4^	21	18.1	202
CXCL11	2.5 × 10^5^	1.3 × 10^−3^	5.2	27.5	135
CXCL12	1.4 × 10^5^	2.1 × 10^−3^	15	18.3	109
K24S/H25S/K27S	1.2 × 10^4^	1.0 × 10^−2^	833.3	2.3	108
CCL2	5.1 × 10^4^	1.5 × 10^−3^	29.4	5.5	80.3
R18A/K19A	NOI*^b^*	NOI	NOI	NOI	NOI
CCL5*^a^*	5.7 × 10^4^	1.1 × 10^−4^	1.9	7.3	265
R44A/K45A/R47A	NPA*^c^*	NPA	NPA	NPA	NPA
CCL7	8.7 × 10^4^	1.6 × 10^−3^	18.4	4.1	41.9
K18A/K19A/K22A	NPA	NPA	NPA	NPA	NPA
CCL19	1.6 × 10^5^	2.8 × 10^−3^	17.5	5.3	53.4
CCL21	7.1 × 10^5^	3.4 × 10^−3^	4.8	18.7	99.5
ΔCT	NPA	NPA	NPA	NPA	NPA
CCL27	2.7 × 10^4^	2.3 × 10^−3^	85.2	8.8	81.9
K25A	8.1 × 10^3^	6.1 × 10^−3^	753.1	6.2	88.5

*^a^* Values for CXCL8 and CCL5 were previously reported in Ref. 30 and are shown here for comparison.

*^b^* NOI means no observable interaction.

*^c^* NPA means no possible analysis due to insufficient signal.

##### GAG-binding Sites of All Chemokines Tested Are Important for Interactions with TSG-6

**FIGURE 2.**
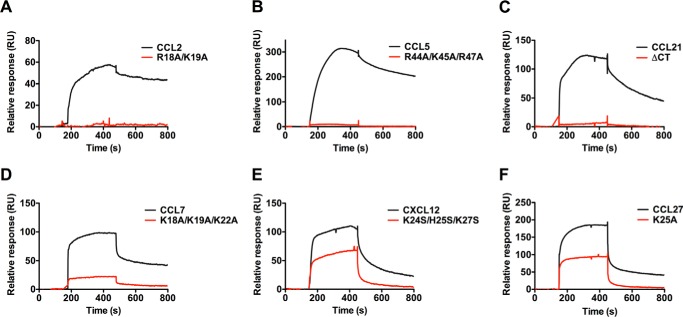
**Chemokines bind Link_TSG6 through their GAG-binding epitopes.** Link_TSG6 was immobilized onto a C1 chip. Chemokines and their corresponding GAG-binding mutants were then passed over the immobilized Link_TSG6 at 40 μl/min, and the resulting interaction was monitored. The response units (*RU*) on the *y* axis reflect the amount of chemokine bound as follows. *A,* CCL2 and R18A/K19A (1000 nm). *B,* CCL5 and R44A/K45A/R47A (200 nm). *C,* CCL21 and C-terminal truncated mutant (200 nm). *D,* CCL7 and K18A/K19A/K22A (1000 nm). *E,* CXCL12 and K24S/H25S/K27S (200 nm). *F,* CCL27 and K25A (1000 nm).

**FIGURE 3.**
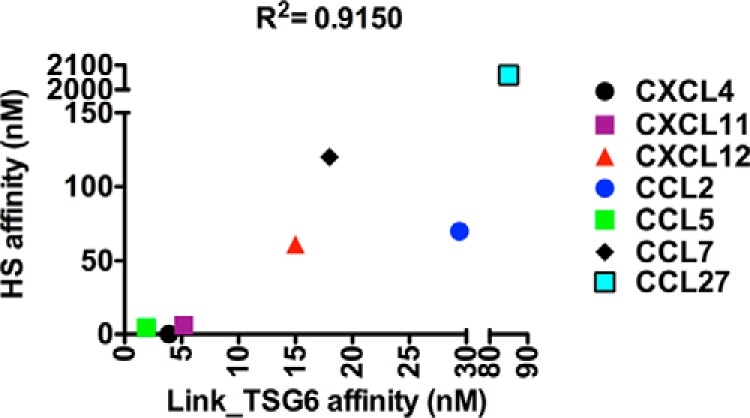
**Correlation between chemokine affinity for Link_TSG6 and HS.** Kinetic affinity estimates for each chemokine binding to immobilized HS (56, 57) or Link_TSG6 are plotted, as calculated using SPR. Data shown are from individual measurements representative of two independent experiments. CXCL8 is excluded from this figure as previous studies did not enable evaluation of a robust affinity estimate for the CXCL8/HS interaction (57).

Previous studies revealed that the GAG-binding region of CXCL8 is involved in its interaction with Link_TSG6 (30), leading us to hypothesize that GAG-binding domains of the chemokines identified above will also be important for Link_TSG6-chemokine complex formation. To this end, we tested whether well characterized GAG-binding deficient chemokine mutants, known to have significantly impaired interactions with heparin or HS, also show a reduced affinity for Link_TSG6. As demonstrated by the sensorgrams in Fig. 2 and the calculated affinities in Table 1, this turned out to be the case. Binding to Link_TSG6 was effectively abolished for the R18A/K19A mutant of CCL2 (59), the R44A/K45A/R47A mutant of CCL5 (60), and a C-terminal deletion mutant of CCL21 (53). Although interaction with Link_TSG6 could be detected with the CCL7 mutant K18A/K19A/K22A (GAG-binding null (56)), there was insufficient signal to calculate a reliable dissociation constant, indicative of a weak interaction. The CXCL12 GAG-binding deficient mutant K24S/H25S/K27S (61) could still bind Link_TSG6 suggesting additional epitopes contribute to the interaction; nevertheless, the affinity was ∼55-fold lower than WT CXCL12. Similarly, the CCL27 K25A mutant displayed an ∼9-fold reduction in affinity for Link_TSG6, and the retention of residual binding is unsurprising as a single point mutation would not be expected to completely eliminate GAG or protein binding. Finally, we could detect only a weak interaction between Link_TSG6 and CCL3 (15 mm (30)), similar to the weak-to-no interaction reported for this chemokine with GAGs (57, 62). Recently, we published apparent affinities determined by SPR for the interaction of HS with CXCL4, CXCL8, CXCL11, CXCL12, CCL2, CCL5, CCL7 (56, 57), and CCL27. A plot of these apparent affinities against Link_TSG6-chemokine affinities demonstrates that there is a positive correlation (Fig. 3). Taken together, these data suggest that the chemokine-binding sites for Link_TSG6 and GAGs overlap, similar to the situation with CXCL8 (30).

##### TSG-6 Blocks Chemokine/GAG Interactions but Not Chemokine/Receptor Interactions

**FIGURE 4.**
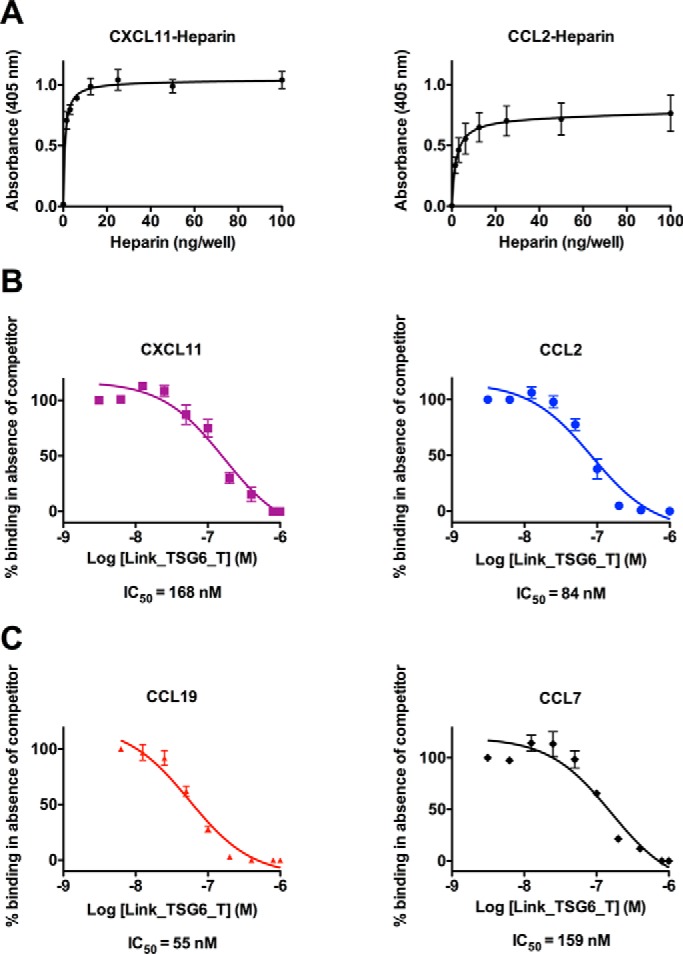
**Link_TSG6 inhibits the interaction of chemokines with heparin.** Chemokine (250 nm) was immobilized onto MaxiSorp plates and incubated with increasing amounts of biotinylated heparin (0–100 ng/well), plates were then washed, and bound heparin was detected (*A*). CXCL11 or CCL2 (250 nm) (*B*) and CCL7 or CCL19 (250 nm) (*C*) were immobilized onto MaxiSorp plates and then incubated with a constant amount of biotinylated heparin (25 ng/well) in combination with increasing concentrations of the Link_TSG6_T mutant (K55A/K69A/K76A) (0–1000 nm), which has reduced heparin binding activity. The amount of bound biotinylated heparin was then detected and plotted as a percentage of the maximum binding observed in the absence of competitor. Data are plotted as the mean of three independent experiments (±S.E.), each undertaken in quadruplicate (*n* = 3) with background signal subtracted.

The overlap between the Link_TSG6- and GAG-binding sites on chemokines suggests that a contributing mechanism by which TSG-6 exerts its anti-inflammatory function *in vivo* may be by blocking chemokine/GAG interactions, which are known to be critical for chemokine function (35–40). To test this hypothesis, an ELISA-based solid phase binding assay was devised. Specifically, CCL2 and CXCL11 (representatives of CC and CXC chemokines, respectively) were separately immobilized onto MaxiSorp plates and incubated with increasing concentrations of heparin, resulting in a saturating interaction between the chemokine and GAG (Fig. 4*A*). These assays were then repeated with immobilized chemokine (CXCL11, CCL2, CCL7, or CCL19) in the presence of a fixed, saturating concentration of heparin in combination with increasing concentrations of the Link_TSG6_T (K55A/K69A/K76A) mutant (Fig. 4, *B* and *C*); this mutant was chosen because it has greatly reduced ability to bind heparin (27) and thereby simplifies the interpretation of the resulting data. In line with the above hypothesis, the Link_TSG6 mutant resulted in a dose-dependent inhibition of the binding of all the chemokines tested to heparin with IC_50_ values estimated to be 168 nm (CXCL11), 84 nm (CCL2), 159 nm (CCL7), and 55 nm (CCL19).

**FIGURE 5.**
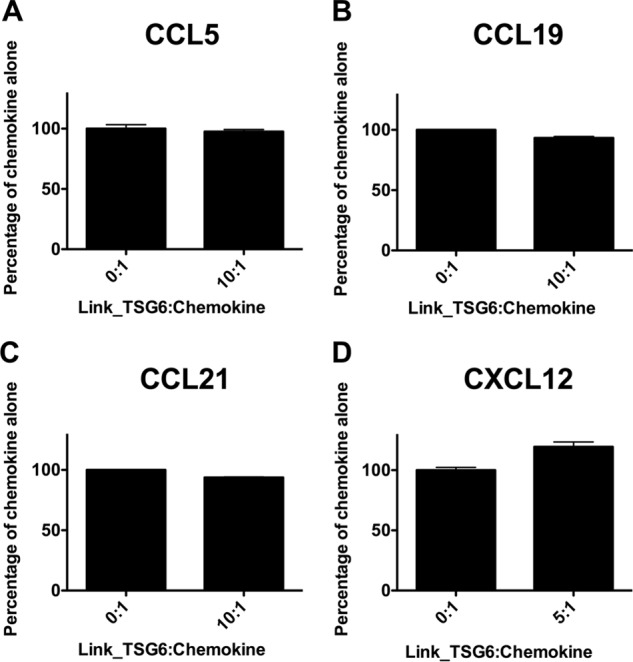
**Pre-incubation with Link_TSG6 has no effect upon CCL5-, CCL19-, CCL21-, or CXCL12-mediated chemotaxis of CCR7-, CCR5-, or CXCR4-expressing cells.** *A–D,* CCL5 (1 nm), CCL19 (50 nm), CCL21 (50 nm), or CXCL12 (1 nm) was added to the bottom chamber of a Transwell system with or without pre-incubation with the indicated molar ratio of Link_TSG6 (Link_TSG6/chemokine). CCR5-expressing (*A*), CCR7-expressing (*B* and *C*), and CXCR4-expressing (*D*) cells were added to the top well of the suspended membrane; following incubation (2 h, 37 °C), the numbers of migrated cells were counted. Data are normalized to the level of migration mediated by chemokine alone and plotted as mean values (±S.E.) from two independent experiments, each undertaken in duplicate (*n* = 2).

To investigate whether Link_TSG6 inhibits the interaction of chemokines with receptor in addition to interactions with GAGs, we conducted bare filter chemotaxis assays in which chemokine was placed in the bottom well of a Transwell apparatus, and L1.2 cells, transfected with appropriate receptor, were placed in the top well. Similar to a previous study where Link_TSG6 had no effect on the migration of CXCR1-expressing cells (30), Link_TSG6 did not affect the migration of CCR5- or CCR7-bearing L1.2 cells toward CCL5, CCL19, or CCL21 or of Jurkat cells that endogenously express CXCR4 toward CXCL12 in the bare filter assay (Fig. 5). These data suggest that although Link_TSG6 directly binds these chemokines, it does not block their interaction with their respective receptors.

##### Link_TSG6 Inhibits Chemokine Presentation on Endothelial Cells and Collagen and Subsequent Chemokine-mediated Cell Adhesion

**FIGURE 6.**
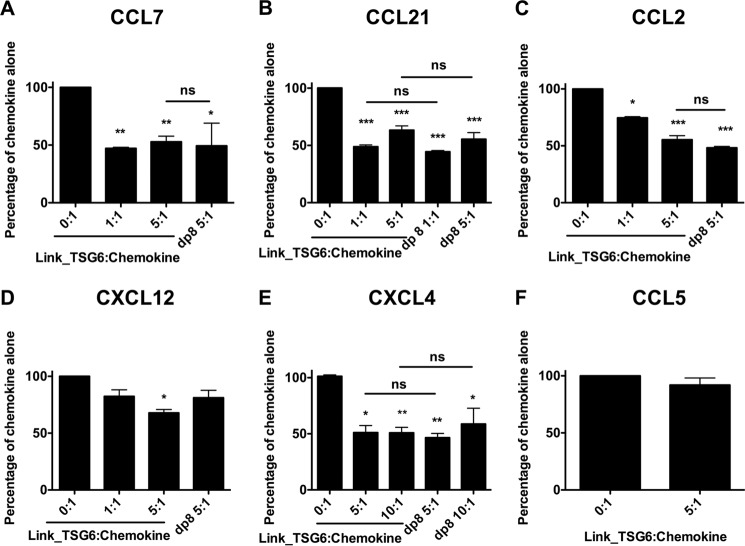
**Pre-incubation of chemokine with Link_TSG6 inhibits subsequent presentation on the endothelial cell surface.** CCL7 (50 nm) (*A*), CCL21 (50 nm) (*B*), CCL2 (50 nm) (*C*), CXCL12 (50 nm) (*D*), CXCL4 (10 nm) (*E*), and CCL5 (10 nm) (*F*) were incubated either alone or in combination with different molar ratios of Link_TSG6 or heparin dp8 (ratios given as Link_TSG6/chemokine or dp8/chemokine) prior to incubation on the endothelial cell surface, followed by washing and detection of bound chemokine. Data are expressed as a percentage of maximal binding of chemokine alone and plotted as mean values (±S.E.) from three independent experiments, each undertaken in duplicate (*n* = 3). *, *p* < 0.05; **, *p* < 0.01; ***, *p* < 0.001 (compared with chemokine-only controls), and *ns* = no significant difference (*p* > 0.05) between samples treated with equivalent molar ratios of Link_TSG6 and dp8, as determined using repeated measures ANOVA analysis with a Bonferroni post hoc test.

The finding that Link_TSG6 interacts with the GAG-binding region of chemokines led us to test whether Link_TSG6 could affect chemokine binding and presentation on endothelial cells, a process known to be mediated by cell-surface GAGs (63, 64). Pre-incubation of Link_TSG6 with six different chemokines resulted in reduced chemokine accumulation on endothelial cells in all cases except CCL5 (Fig. 6). The potency of its inhibitory trend was chemokine-specific as a 1:1 Link_TSG6/chemokine molar ratio was sufficient to significantly reduce the endothelial cell accumulation of CCL7 (down to 50% of the level of chemokine alone), CCL21 (50% of chemokine alone), and CCL2 (∼75 and ∼55% of chemokine alone at a 1:1 and 5:1 ratio, respectively, Fig. 6, *A–C*). However, CXCL12 and CXCL4 required a 5-fold molar excess of Link_TSG6 to reduce accumulation to 68 and 51% of chemokine alone, respectively (Fig. 6, *D* and *E*, respectively); lower ratios of Link_TSG6/CXCL4 had no effect on endothelial binding of the chemokine (data not shown). No inhibition of CCL5 was observed at a 5:1 ratio of Link_TSG6 to chemokine (Fig. 6*F*). Importantly, when the experiment was performed using heparin dp8 instead of Link_TSG6 in the same molar excess that caused reduced chemokine binding with Link_TSG6, a similar inhibition of chemokine accumulation on the endothelial cells was observed for CCL7, CCL21, CCL2, and CXCL4 (Fig. 6, *A–C* and *E*, respectively). In the case of CXCL12, pre-incubation with either heparin dp8 or Link_TSG6 had similar inhibitory effects on chemokine accumulation, although in contrast to Link_TSG6, the effect of heparin did not reach significance (Fig. 6*D*).

**FIGURE 7.**
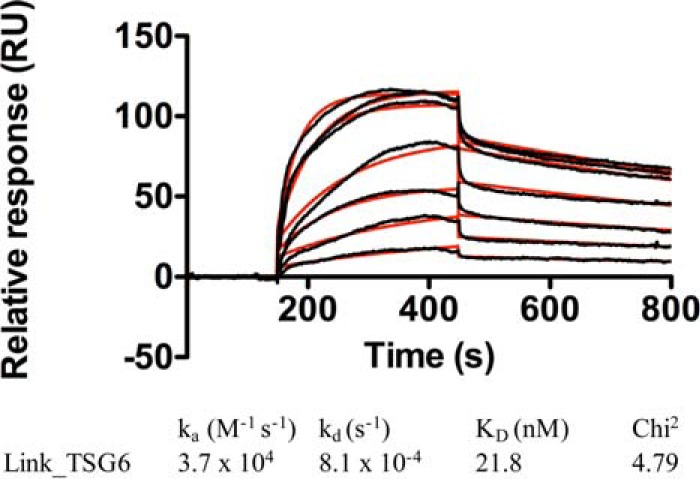
**Link_TSG6 binds immobilized heparin.** Heparin was immobilized onto a C1 BIAcore chip before Link_TSG6 was passed over in running buffer at a range of concentrations (750, 500, 400, 250, 200, 100, and 50 nm). Rates of association (*k_a_*) and rates of dissociation (*k_d_*) were used to calculate overall affinity (*K_D_* = *k_d_*/*k_a_*). The quality of the fit to a 1:1 Langmuir model is given by χ^2^ values calculated by application of this model, where χ^2^ <10 is indicative of a good fit.

**FIGURE 8.**
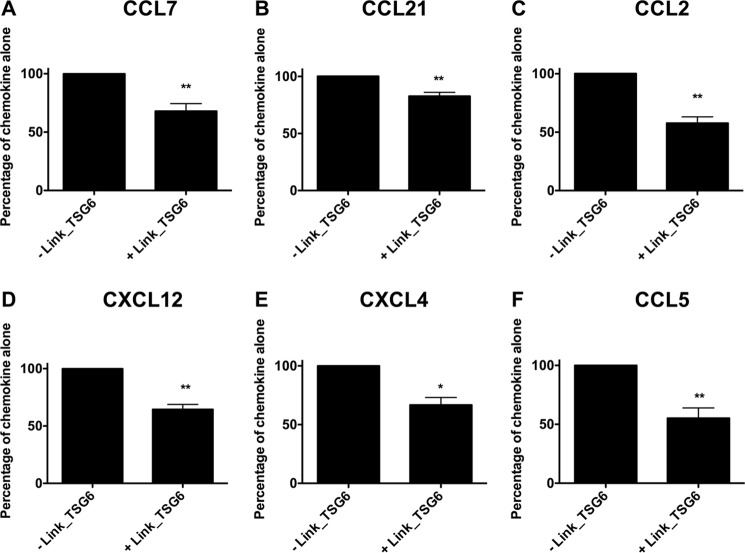
**Pre-incubation of Link_TSG6 on the endothelial cell surface inhibits subsequent chemokine presentation.** Endothelial monolayers were incubated with/without Link_TSG6 (500 nm) followed by washing and addition of CCL7 (50 nm) (*A*), CCL21 (50 nm) (*B*), CCL2 (50 nm) (*C*), CXCL12 (50 nm) (*D*), CXCL4 (10 nm) (*E*), and CCL5 (10 nm) (*F*) to the endothelial cell surface; after washing, the level of bound chemokine was determined. Data are expressed as a percentage of maximal binding of chemokine alone, plotted as mean values (±S.E.) from three independent experiments, each undertaken in duplicate (*n* = 3). *, *p* < 0.05; **, *p* < 0.01 (compared with chemokine-only controls), as determined using Student's *t* test.

Link_TSG6 was previously demonstrated to have a moderate affinity (∼3 μm) interaction with dp8 heparin by isothermal titration calorimetry (27). By comparison and as shown by the SPR data in Fig. 7, Link_TSG6 binds unfractionated heparin (average molecular mass = 15 kDa, dp 10–80) with higher affinity (∼22 nm); this is not surprising because larger GAGs have been observed to have higher affinity for chemokines (62). This affinity is in the range of chemokine/heparin interactions (57), which prompted us to investigate whether Link_TSG6 could also affect chemokine/endothelial cell surface binding when incubated with the cells prior to introduction of chemokine. This experimental design specifically addresses an alternative mechanism of inhibition, where Link_TSG6 binds endothelial GAGs, and thereby affects subsequent chemokine/GAG interactions. In this experiment, Link_TSG6 inhibited the accumulation of CXCL4 (33% inhibition), CXCL12 (36% inhibition), CCL2 (42% inhibition), CCL7 (32% inhibition), CCL21 (17% inhibition), and CCL5 (45% inhibition) on endothelial cell surfaces (Fig. 8). These data suggest that Link_TSG6 can prevent endothelial presentation via a direct interaction with chemokines and also by masking or limiting available cell-surface GAGs.

**FIGURE 9.**
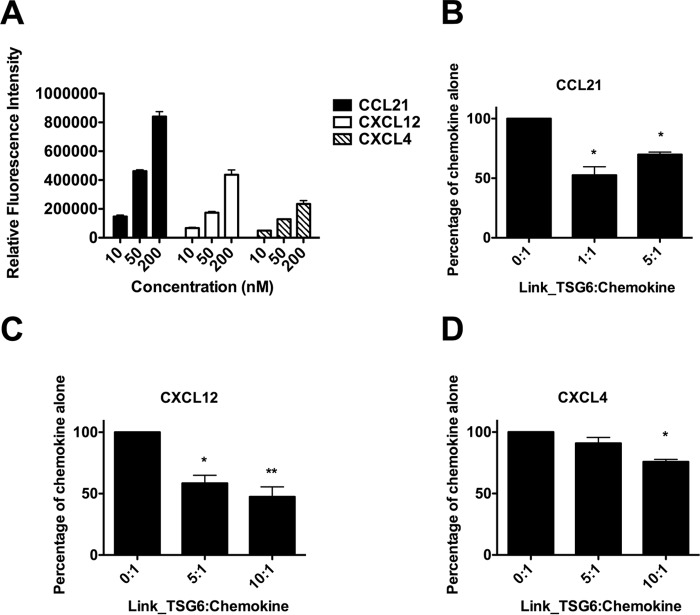
**Pre-incubation of chemokine with Link_TSG6 inhibits subsequent presentation on collagen.** Biotinylated CCL21, CXCL12, or CXCL4 were incubated at different concentrations (10, 50, or 200 nm) on wells pre-coated with collagen, and the amounts bound following washing were detected using labeled streptavidin (*A*). The biotinylated chemokines CCL21 (50 nm) (*B*), CXCL12 (50 nm) (*C*), or CXCL4 (10 nm) (*D*) were incubated either alone or in combination with different molar ratios of Link_TSG6 (ratios given as Link_TSG6/chemokine) prior to incubation on the collagen-coated surfaces, followed by detection as before. Data are expressed as total binding (relative fluorescence intensity) (*A*) or as a percentage of maximal binding of chemokine alone (*B–D*), plotted as mean values (±S.E.) from two independent experiments, each undertaken in duplicate (*n* = 2). *, *p* < 0.05; **, *p* < 0.01 (compared with chemokine-only controls), as determined using repeated measures ANOVA analysis with a Bonferroni post hoc test.

Chemokines are found abundantly in the ECM (31), and TSG-6 is known to function in the ECM (5, 9, 10). Thus, following our observations that Link_TSG6 can inhibit chemokine presentation on endothelial cells, we investigated whether it inhibits chemokine binding to collagen, an important component of the ECM. In agreement with previous studies describing chemokine/collagen interactions (65, 66), we observed that CCL21, CXCL12, and CXCL4 bind to collagen-coated surfaces (Fig. 9*A*). Notably, pre-incubation of these chemokines with Link_TSG6 resulted in dose-dependent inhibition of their binding to collagen (Fig. 9, *B–D*). These findings suggest TSG-6 may function as a general modulator of chemokine presentation on endothelial cell HS and extracellular matrix components, including GAGs and collagen.

**FIGURE 10.**
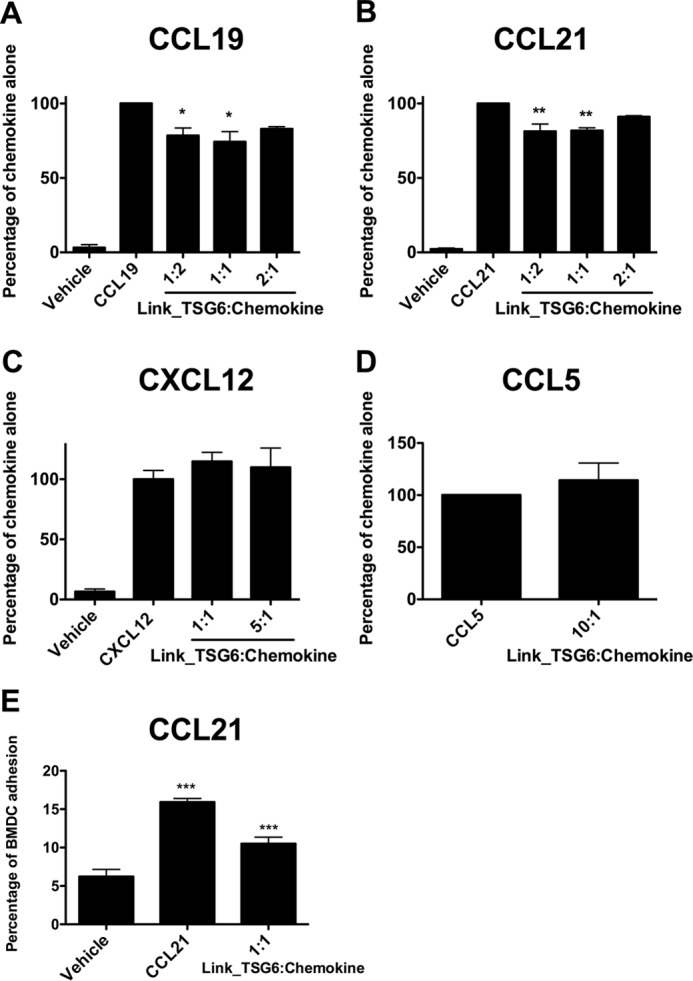
**Pre-incubation with Link_TSG6 inhibits CCL19- and CCL21-mediated but not CXCL12- or CCL5-mediated transendothelial migration of CCR7-, CXCR4-, or CCR5-expressing cells, and CCL21-mediated adhesion of BMDCs.** CCL19 (50 nm), CCL21 (50 nm), CXCL12 (1 nm), or CCL5 (1 nm) was added to the bottom chamber of a Transwell with or without pre-incubation with the indicated molar ratios of Link_TSG6 (Link_TSG6/chemokine) and CCR7- (*A* and *B*), CXCR4- (*C*), or CCR5 (*D*)-expressing cells were added to the top well in the presence of an endothelial monolayer on the suspended membrane; following incubation (2 h, 37 °C), the numbers of migrated cells were counted. Data were normalized to the level of migration mediated by chemokine alone, plotted as mean values (±S.E.) from three independent experiments, each undertaken in duplicate (*n* = 3). *, *p* < 0.05; **, *p* < 0.01 (compared with chemokine-only controls), as determined using repeated measures ANOVA analysis with a Bonferroni post hoc test. CCL21 (10 nm), pre-incubated at a 1:1 molar ratio with Link_TSG6, was incubated with SV-LECs (30 min, 37 °C) followed by addition of BMDCs. BMDC adhesion (percentage of total maximal signal) is plotted as mean values for triplicate wells (± S.D.) from a representative data set of six independent experiments (*E*). ***, *p* < 0.001 for CCL21 alone compared with vehicle control or for Link_TSG6/chemokine compared with CCL21 alone, as determined using a one-way ANOVA analysis with a Bonferroni post hoc test.

Link_TSG6 did not inhibit the interaction of chemokines studied herein with their receptors as shown by its inability to affect cell migration in an *in vitro* bare filter chemotaxis assay. However, because Link_TSG6 inhibits presentation on endothelial GAGs, we questioned whether it would inhibit transendothelial cell migration, a process that requires transport of chemokine across the endothelial layer and can involve GAG-dependent transcytosis (30, 64, 67). Moreover, in a previous study, Link_TSG6 significantly inhibited transendothelial migration of cells toward CXCL8, which correlated with impaired CXCL8 transcytosis and impaired presentation of the chemokine on the apical surface (30). In this study, it inhibited CCL19- and CCL21-mediated transmigration of L1.2/CCR7 cells, albeit modestly, at both 1:2 (∼22 and ∼19% inhibition, respectively) and 1:1 (∼26 and ∼18% inhibition, respectively) Link_TSG6/chemokine ratios (Fig. 10). As transcytosis has been suggested to account for only 10% of chemokine transport, with pericellular transport by diffusion of chemokine through gaps between endothelial cells accounting for ∼90% (64), it is not surprising that only modest inhibition was observed. Additionally, we did not observe any inhibitory effects on CXCL12- or CCL5-mediated transmigration of receptor-bearing Jurkat or L1.2 cells, respectively (Fig. 10, *C* and *D*), which suggests that these chemokines are transported predominantly by a pericellular route. Although only modest inhibition of CCL21-mediated transendothelial cell migration was observed, we sought to determine whether Link_TSG6 could disrupt other steps in this process. Specifically, inhibition of dendritic cell adhesion to the endothelium was tested, given the established reduction in CCL21 accumulation on the apical surface of endothelial cells in the presence of Link_TSG6 (Fig. 6*B*). Pre-incubation of CCL21 with Link_TSG6 at a 1:1 molar ratio significantly reduced CCL21-mediated BMDC adhesion to close to control levels (with a mean value of 40% inhibition and a range of 23–71% inhibition across replicate experiments) (Fig. 10*E*). This finding provides additional evidence for a biologically relevant consequence of TSG-6-mediated regulation of chemokine function.

## Discussion

### 

#### 

##### TSG-6 Link Module Mediates Binding to Multiple Chemokines

Given that Link_TSG6 inhibits neutrophil recruitment and associated inflammation by blocking the function of CXCL8, we set out to test whether it inhibits the function of other chemokines. These studies were also motivated by its broad anti-inflammatory effects in several disease models where a wide range of other chemokines and cell types play a role (4, 11–19, 22, 23). Indeed, we demonstrated that Link_TSG6 binds to multiple chemokines from both the CC and CXC families, including CXCL4, CXCL11, CXCL12, CCL2, CCL5, CCL7, CCL19, CCL21, and CCL27. This group not only includes chemokines from two of the four chemokine subfamilies but also those classified as inflammatory (CXCL4, CXCL11, CXCL12, CCL2, CCL5, and CCL7) *versus* homeostatic (CCL19 and CCL21) (68, 69).

The fact that TSG-6 binds to most chemokines tested raises the question as to the likelihood that it would be present in the same location as these chemokines *in vivo*. TSG-6 expression during inflammation is well established (5, 8), and it is known to be secreted by inflammatory cells such as peripheral blood mononuclear cells (1, 7, 70), neutrophils (71), mast cells (72), and macrophages (71, 73) in response to signals, including LPS and TNF (1, 7, 70, 71). TSG-6 is also produced by stromal cells such as fibroblasts (7, 74) and human umbilical vein endothelial cells (71). Likewise, many chemokines are associated with inflammation (32, 34) and are expressed by a similar range of inflammatory (45, 76–78) and endothelial (79, 80) cells. Unlike inflammatory chemokines, CCL19 and CCL21 are associated with lymphatic trafficking of dendritic cells via CCR7 (81); thus, their expression is more limited to cells in lymphatic vessels. Nevertheless, the fact that monocyte-derived dendritic cells produce TSG-6 in response to LPS (71) suggests that lymphatic dendritic cells could also produce TSG-6. Similarly, human umbilical vein and microvascular endothelial cells have been shown to produce TSG-6 (71, 82), and TSG-6 has been implicated in the function of HA binding to the LYVE-1 receptor on lymph vessel endothelial cells (83). Overall, it seems likely that TSG-6 and chemokines would be co-expressed *in vivo* at sites of inflammation and in the lymphatic system.

##### TSG-6 Interacts with Chemokines through Their GAG-binding Sites and Inhibits Their Binding to GAGs and Endothelial Cell Surfaces

**FIGURE 11.**
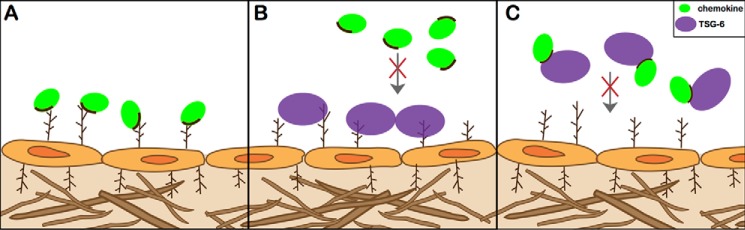
**Model of TSG-6 inhibition of chemokine/GAG interactions.** *A,* chemokines (*green*) bind to GAGs (*branched structures*) present on endothelial cells (shown) and within the ECM (not shown) via GAG-binding sites on the chemokine (depicted as *dark line*). Integral to their function, chemokine/GAG interactions enable the retention and accumulation of chemokines on cell surfaces, which leads to the formation of chemokine gradients involved in directing cell migration. In inflammatory settings, TSG-6 (*purple*) is up-regulated and inhibits chemokine function by blocking the cell surface presentation of chemokines (*B* and *C*). One mechanism for how TSG-6 can exert its inhibitory effects is by directly binding to GAGs on the cell surface, thus limiting available GAG for chemokine interactions (*B*). Additionally, TSG-6 binds chemokines directly through their GAG-binding domains, thereby blocking chemokine interactions with GAG by competing for a common binding surface (*C*).

In previous studies of CXCL8 (30), it was shown that Link_TSG6 does not exert its inhibitory effects by disrupting chemokine/receptor interactions (except at high micromolar concentrations in the case of CXCR2). Instead, it primarily blocks the interaction of CXCL8 with GAGs (27), which indirectly affects cell migration (35–40). Chemokine/GAG interactions are thought to be required for the formation of chemokine gradients on cell surfaces and in the ECM, as demonstrated for CCL21 in the context of dendritic cell recruitment (40). Moreover, GAG-binding deficient mutants of CXCL8, CXCL12, CCL2, CCL5, and CCL7 all show a significantly impaired ability to recruit cells *in vivo*, despite their capacity to promote cell migration in bare filter chemotaxis assays where GAG binding is not required (35–37, 39). Given the prior data on CXCL8, we hypothesized that TSG-6 would also target the GAG-binding sites of the chemokines identified as ligands in this study. Indeed, we showed that mutation of GAG-binding residues in chemokines greatly reduced the affinity of Link_TSG6. Thus, it was not surprising to find that incubation of Link_TSG6 with most of the tested WT chemokines inhibited their presentation on endothelial cell surfaces (Fig. 11, *A* and *C*). Furthermore, Link_TSG6 was as effective as heparin at inhibiting endothelial presentation (when used at an equivalent molar ratio), and it also blocked binding of chemokines to collagen, an important component of the ECM.

The exception to these findings was the lack of inhibition of CCL5 binding to endothelial cells following pre-incubation with Link_TSG6, despite the high affinity interaction between the two. The mechanistic reason for the anomalous behavior of CCL5 is unknown; however, CCL5 is unique in forming large stable polymers in solution, and it has a very high affinity for cell-surface GAGs because of avidity effects from multiple GAG-binding sites on its surface (57). Although speculative, it may be that upon pre-incubation with CCL5, TSG-6 is unable to mask all GAG-binding sites on the CCL5 polymer and that the remaining GAG sites are still permissive to its interaction with cell-surface GAGs. However, when TSG-6 is pre-incubated with the endothelial surface, it may be a more effective mechanism for blocking the subsequent binding of CCL5 by sterically preventing the formation of high affinity GAG-CCL5 polymer complexes that would normally occur. Further studies will be required to test this possibility.

In addition to inhibiting the binding of chemokines to endothelial cells by interacting with the GAG-binding epitopes on chemokines, we showed that TSG-6 can directly interact with and thereby mask cell-surface GAGs (Fig. 11, *A* and *B*). In support of these findings, it is well established that the Link_TSG6 interacts with a wide variety of GAGs, including chondroitin sulfate (CS), dermatan sulfate, heparin/HS, and HA (27, 28). Furthermore, in this study we demonstrate that Link_TSG6 binds to heparin with an affinity (∼20 nm) that is similar to its affinity for several chemokines (56, 57). We also showed that the affinities of chemokines for Link_TSG6 strongly correlate with their affinities for HS. This “matching” of binding strengths would be expected to allow TSG-6 to modulate chemokine binding to GAGs in a highly concentration-dependent manner.

The ability of the TSG-6 Link module to dimerize when bound to heparin and CS (27, 84) may contribute to its potency in sequestering chemokine-binding sites on GAGs through an avidity effect. Cross-linking of GAGs by TSG-6 may also affect the accessibility or conformation of chemokine-binding sites on GAGs. Finally, binding of the CUB_C domain to fibronectin in the context of full-length TSG-6 (85) may provide additional tethers that further strengthen its interactions with GAGs, collagen, and/or other ECM substrates. Collectively, these mechanisms for sequestering chemokine-binding sites on endothelial and ECM GAGs may contribute to the broad spectrum activities of TSG-6, enabling it to affect not only the chemokines investigated here but any chemokine whose function is dependent on GAG binding.

By inhibiting the presentation of chemokines on cell-surface GAGs and collagen, Link_TSG6 is predicted to cause the disruption of chemokine gradients, resulting in impaired cell migration (40). However, the *in vitro* migration assays used in this study are inadequate for testing this hypothesis because, apart from a potentially small contribution of GAG-mediated transcytosis on cell migration, there is little or no dependence of transendothelial migration on GAG interactions. This is due to the fact that Transwell migration assays automatically establish a chemokine gradient by the separation of chemokine and cells between the two chambers, eliminating the need for GAGs. Furthermore, sample confinement in the wells and lack of shear forces/flow prevent rapid dissipation of chemokines that might otherwise happen *in vivo* in the absence of GAG interactions (35). More complex *in vitro* (under flow) or *in vivo* assays will be required to further probe the mechanistic basis for the anti-inflammatory properties of TSG-6 with respect to how it affects gradient formation of chemokines and subsequently cell migration, similar to previous studies with GAG binding-deficient chemokines (35–37, 39).

##### Insights into the Recognition between Chemokines and TSG-6

**FIGURE 12.**
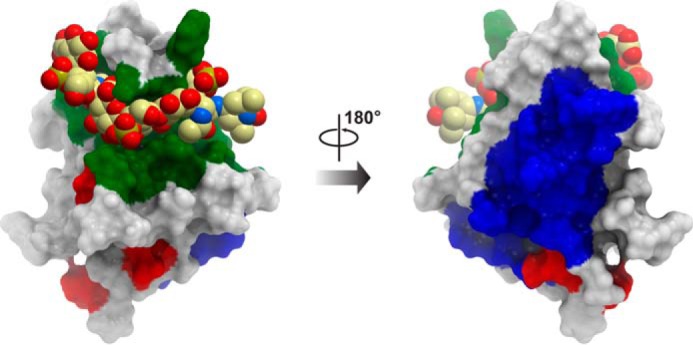
**Interaction sites for CS/HA, heparin, and potential binding sites for chemokines on Link_TSG6.** Structure of a Link_TSG6 complex with CS (Protein Data Bank code 2N40) is shown, where Link_TSG6 is represented as a surface mesh, and CS is shown as a space-filling model. The *green* surface of Link_TSG6 highlights the aromatics that define the CS/HA-binding site (9, 84) and could provide a binding site for chemokines through their GAG-binding epitopes. The *blue* surface highlights the basic residues that define the heparin-binding site as determined previously (75), which is not compatible with chemokine binding. The *red* surface highlights Glu residues that could provide a binding site for chemokines through their GAG-binding epitopes.

In this study, we showed that TSG-6 binds to all chemokines tested through their GAG-binding sites. The high resolution molecular details of how it does so remains an important question because chemokines are overall basic proteins, but TSG-6 is basic as well (pI ∼9.48 (84)). Previously, it was shown that a mutant of Link_TSG6 (Link_TSG6_T), with reduced capacity to bind heparin (27), was still capable of binding to CXCL8 and inhibiting both its interaction with GAGs and transendothelial cell migration toward CXCL8 (30). Similarly, in this study, we showed that this Link_TSG6 mutant could inhibit CXCL11, CCL2, CCL7, and CCL19 interactions with heparin. These findings suggest that the heparin-binding site on Link_TSG6 (Fig. 12) does not overlap with its binding site for CXCL8 and the above chemokines, which may very well be the case for other chemokines. This would not be surprising because the heparin-binding surface of Link_TSG6 is composed of basic residues, making it incompatible with chemokine GAG-binding sites, which are also defined by clusters of Arg, Lys, and His (86, 87). Instead, the interaction with the GAG-binding domains of chemokines may be mediated by negatively charged amino acids in Link_TSG6 (Fig. 12). Alternatively, as the binding site on Link_TSG6 for HA/CS is non-overlapping with heparin and enriched with aromatic residues, it is possible that it could provide a binding site for chemokines (Fig. 12). In this case, favorable cation/π interactions (88, 89) between the Link_TSG6 aromatic residues and Lys/Arg residues in the chemokine GAG-binding epitopes could contribute to complex formation. Identification of the chemokine-binding sites on TSG-6 will be the subject of future studies.

The present data also suggest that, although the binding sites for chemokines and heparin on Link_TSG6 do not overlap, it is probable that chemokine and heparin (and likely HS) do not simultaneously bind the Link module (Fig. 11). This is because Link_TSG6 blocks cell surface presentation of chemokines, and if GAG and chemokine could both bind to the Link module, then the opposite effect might be expected. This suggests that an allosteric mechanism may prevent simultaneous binding of GAG and chemokine, as has been suggested for the competing interactions of HA and heparin for their distinct binding sites on Link_TSG6 (27, 84). Alternatively, if chemokines bind to the HA/CS-binding site of TSG-6 (27, 84), then binding of chemokine and these GAGs to TSG-6 would simply be competitive and mutually exclusive.

In addition to basic epitopes defining the GAG-binding sites of chemokines, many chemokines oligomerize by themselves, and the oligomers are stabilized by GAGs (56, 90–93). Furthermore, oligomerization plays a critical role in the affinity of chemokines for heparin, HS, and CS (39, 56, 57, 94). Whether or not chemokine oligomerization affects binding to Link_TSG6 is not yet clear and will also be the subject of future studies.

##### TSG-6 Is a Broad Spectrum Chemokine-binding Protein

We previously identified TSG-6 as the first known mammalian soluble chemokine-binding protein (30) and can now further define it as a broad spectrum chemokine-binding protein that interacts with multiple chemokines via their GAG-binding domains. Thus, the cumulative abilities of TSG-6 to inhibit chemokine interactions with GAGs as well as collagen, to exert inhibitory effects in the ECM and on cell surfaces, and to directly bind chemokines and GAGs together create a robust mechanism by which TSG-6 is able to modulate the function of many chemokines with potentially significant anti-inflammatory consequences (11–14, 16–19, 23). In fact, TSG-6 may represent a general regulator of heparin/HS-binding proteins; in addition to 10 chemokines, TSG-6 interacts with seven heparin/HS-binding bone morphogenetic proteins (95), at least in part, via its Link module. How a single protein can bind so many other proteins is reminiscent of viral chemokine-binding proteins that promote virulence by sequestering chemokines and suppressing the immune response (96). For example, the chemokine-binding protein M3, produced by γ-herpesvirus 68, can bind to both receptor- and GAG-binding domains of chemokines (97), thereby inhibiting chemokine function by two separate mechanisms. E163 from poxvirus is similar to TSG-6 in that it interacts with chemokines through their GAG-binding epitopes and also binds directly to GAGs, but it does not inhibit interactions of chemokines with their receptors (46). The similarity of TSG-6 with these various chemokine-binding proteins underscores its role as a pluripotent anti-inflammatory mediator of chemokine function. Its ability to interact with so many chemokines may add to its known functions in the ECM and explain some of its protective effects in models of inflammatory disease (11–14) and its more recently described role in protection against inflammatory damage mediated by human mesenchymal stem cells (4, 16–18, 23). Inhibition of the presentation of chemokines on cell surface and ECM GAGs would be expected to impair leukocyte migration *in vivo* due to the lack of an immobilized chemokine gradient for the cells to follow (40). Thus, TSG-6 expression may provide a novel mechanism whereby cellular presentation of chemokines can be regulated and finely tuned.

## Author Contributions

D. P. D. purified chemokine, performed SPR, solid phase binding assays, cell migration assays, and endothelial/collagen chemokine binding assays and assisted in drafting the manuscript. C. L. S. purified chemokine, optimized endothelial chemokine binding assays and cell migration assays, generated Fig. 11, provided technical and intellectual input, and assisted in drafting the manuscript. S. C. J. performed the BMDC adhesion assays. E. V. performed solid phase competition binding assays. M. M. F. directed the BMDC adhesion assays. C. M. M. co-directed the studies and assisted in drafting the manuscript. A. J. D. co-directed the studies and assisted in drafting the manuscript. T. M. H. directed the study and coordinated the writing of the manuscript.

## Footnotes

4 The abbreviations used are:

GAG: glycosaminoglycan
SPR: surface plasmon resonance
Link_TSG6: recombinant Link module from human TSG-6
CXCL: CXC-type chemokine ligand
ECM: extracellular matrix
CCL: CC-type chemokine ligand
HS: heparan sulfate
CS: chondroitin sulfate
HA: hyaluronan
BMDC: bone marrow-derived dendritic cell
ANOVA: analysis of variance
SV-LEC: murine lymphatic endothelial cell.
